# Cardiovascular-kidney-metabolic syndrome and cancer risk: Evidence from multi-omics analysis results

**DOI:** 10.1515/jtim-2026-0072

**Published:** 2026-08-24

**Authors:** Shengtao Hu, Qianxi Ye, Rui Yang, Liang Ma, Jiayuan Wu, Zhijun Dai

**Affiliations:** Department of Breast Surgery, The First Affiliated Hospital, College of Medicine, Zhejiang University, Hangzhou, Zhejiang Province, China; Department of Cardiovascular Surgery, the First Affiliated Hospital, College of Medicine, Zhejiang University, Hangzhou, Zhejiang Province, China; School of Nursing, Zhejiang Chinese Medical University, Zhejiang Province, Zhejiang, China; Clinical Research Service Center, Affiliated Hospital of Guangdong Medical University, Zhanjiang, Guangdong Province, China

## To the editor

Cardiovascular-kidney-metabolic (CKM) Syndrome, introduced by the American Heart Association in 2023, represents a newly defined systemic disorder characterized by the progressive and interconnected deterioration of cardiovascular, renal, and metabolic systems.^[1]^ The CKM framework highlights the cumulative and interactive nature of multi-organ dysfunction. Concurrently, cancer, the second leading cause of death globally, accounts for approximately 10 million deaths annually.^[2]^ Both diseases share pathogenic mechanisms.^[3]^

However, the relationship between CKM syndrome and cancer remains unclear. Research has demonstrated that cardiovascular disease, chronic kidney disease, and metabolic disorders individually correlate with elevated cancer risk, though these conditions have typically been studied separately.^[3]^ In fact, patients frequently present with multiple clinical diseases and metabolic abnormalities simultaneously, creating an intricate network of comorbidities. The systemic dysfunction spanning the cardiovascular, renal, and metabolic domains in CKM syndrome may act synergistically through shared pathological pathways to exacerbate DNA damage and genomic instability,^[3, 4, 5]^ thereby creating conditions more favorable for oncogenesis than any individual disorder alone.

In this study, we utilized three multinational studies—UK Biobank (UKBB), National Health and Nutrition Examination Surveys (NHANES), and China Health and Retirement Longitudinal Study (CHARLS)—to examine the association between CKM syndrome and cancer risk. Mendelian randomization (MR) was used to assess potential genetic associations. We then identified plasma proteomic and metabolic characteristics of advanced CKM syndrome and enriched potential biological processes. Mediation analysis was conducted to quantitatively evaluate potential mediating pathways.

Baseline characteristics are shown in Supplementary Table S1. In UKBB (*n* = 182, 140; median follow-up 13.6 years), 18,390 cancer events occurred. In NHANES (*n* = 18, 079), 1314 cancer events were observed. In CHARLS (*n* = 7944; median follow-up 6.9 years), 133 cancer events occurred. Across UKBB and NHANES, advancing CKM syndrome stage was associated with progressively higher overall cancer risk. As Stage 3 classification was based on predicted risk rather than direct measures of sub-clinical disease, some degree of non-differential misclassification may have occurred, potentially attenuating the observed associations. Additionally, differences in outcome ascertainment—registry-confirmed cancer in UKBB versus self-reported diagnoses in NHANES and CHARLS—may have contributed to variation in effect estimates across cohorts.

**Figure 1:**
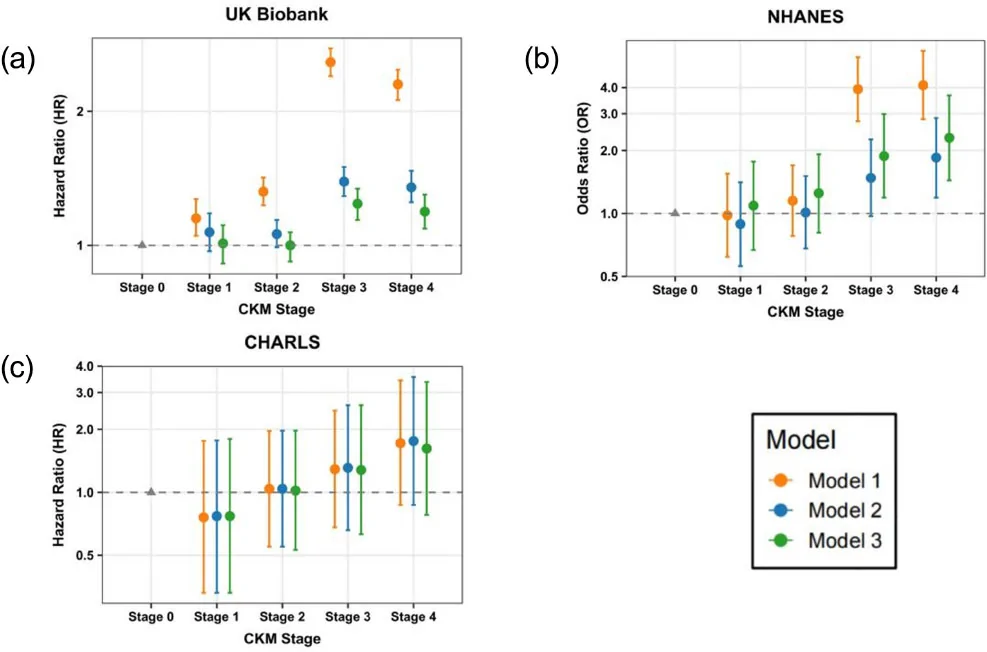
Multivariate adjusted risk ratio for CKM syndrome with risk of overall cancer. (a) Multivariate adjusted hazard ratios for CKM syndrome with risk of overall cancer in UKBB. (b) Multivariate adjusted odds ratios for CKM syndrome with risk of overall cancer in NHANES. (c) Multivariate adjusted hazard ratios for CKM syndrome with risk of overall cancer in CHARLS (results from CHARLS should be interpreted with caution due to the limited number of cancer events, which may have reduced statistical power). Model 1 was only adjusted for CKM stage; Model 2 was additionally adjusted for sex and age; Model 3 were fully adjusted for sex, age, ethnicity (NHANES only), socioeconomic status, marital status, education level, BMI, levels of physical activity (only in UKBB and NHANES), smoking status, alcohol consumption, diet points (only in UKBB and NHANES). CKM: cardiovascular-kidney-metabolic; UKBB: UK Biobank; NHANES: National Health and Nutrition Examination Survey; CHARLS: China Health and Retirement Longitudinal Study.

In fully adjusted models, advanced CKM stages remained independently associated with cancer risk (Figure 1). In UKBB, Stage 3 and Stage 4 were associated with increased cancer risk compared with Stage 0 (Stage 3: hazard ratio [HR] 1.24, 95% confidence interval [95% CI] 1.14–1.34, *P* < 0.001; Stage 4: HR 1.19, 95% CI 1.09–1.30, *P* < 0.001). In NHANES, the corresponding associations were stronger (Stage 3: odds ratio [OR] 1.88, 95% CI 1.19–2.99, *P* = 0.008; Stage 4: OR 2.30, 95% CI 1.44–3.67, *P* < 0.001). Cancer risk increased with the accumulation of CKM components. Notably, coexistence of CVD and CKD conferred the highest risk in both cohorts, supporting a significant additive effect of CKM burden on cancer development. In fully adjusted models, CKM Stage 3–4 was significantly associated with multiple site-specific cancers in UKBB and NHANES compared with Stage 2. Stratified analyses suggested heterogeneity across populations. In UKBB, the association of advanced CKM syndrome (Stages 3–4) with cancer risk was stronger in smokers, and remained significant only among alcohol drinkers. In NHANES, similar patterns were observed, with stronger associations in smokers and alcohol drinkers; the association also appeared more pronounced in younger participants and females. In CHARLS, no statistically significant associations were observed, likely reflecting the limited number of cancer events and the resulting insufficient statistical power.

Genetically predicted CKM was not significantly associated with risk of any cancer type. Differentially expressed proteins were used to construct a protein risk score (ProRS) for advanced CKM. Pathway enrichment analyses indicated that down-regulated proteins were mainly involved in coagulation and lipid pathways, whereas up-regulated proteins were enriched in immune and inflammatory processes. Metabolic data analysis revealed significant alterations in lipid and lipoprotein profiles in advanced CKM, consistent with the bio-enrichment results for downregulated proteins. Key findings include substantial reductions in various lipid and lipoprotein subclasses. Differentially expressed metabolic components were used to construct a metabolic risk score (MetRS) for advanced CKM. Higher MetRS and ProRS are both associated with a higher overall cancer risk (HR_ProRS_ = 1.11, *P* < 0.001; HR_MetRS_ = 1.07, *P* < 0.001). In UKBB, all inflammatory markers demonstrated a significant mediating effect on the association between CKM syndrome and overall cancer risk, with neutrophil count contributing the highest mediation proportion (11.13%). MetRS explained 16.60% of the total effect. In contrast, in NHANES, all inflammatory markers except neutrophil count showed significant positive mediation effects, with leukocyte count accounting for the largest mediation proportion (4.91%).

Previous research has primarily examined individual CKM components in relation to cancer risk, often yielding heterogeneous findings and inconsistent results in Mendelian randomization (MR) analyses.^[6,7]^ Our findings suggest that CKM, as an integrated multidimensional construct, better captures multi-morbidity-related cancer susceptibility than its individual components alone. As the MR analyses did not identify significant or consistent genetic causality, the observed associations are unlikely to be driven primarily by shared lifelong genetic liability. Rather than contradicting the observational findings, this discrepancy suggests that the CKM–cancer relationship may be more strongly related to the acquired and progressive pathophysiological state of advanced CKM syndrome than to inherited genetic susceptibility. Genetic instruments from existing GWAS may not fully capture the complex multisystem nature of CKM syndrome and may also be affected by residual horizontal pleiotropy. In contrast, observational analyses reflect cumulative environmental and behavioral exposures, including chronic inflammation and metabolic dysfunction, that develop over time^[3]^ and may promote carcinogenesis through epigenetic alterations and changes in the tissue microenvironment.^[8]^ Consistently, our multi-omics and mediation analyses showed that advanced CKM was marked by inflammatory activation and lipid–metabolic dysregulation, and that these pathways significantly mediated the association with cancer risk.

Notably, the association between CKM syndrome and cancer risk is most pronounced in its late stages. Proteomic data show that proteins upregulated in advanced CKM are enriched in prototypical pro-inflammatory and immune response pathways.^[9]^ In parallel, both proteomic and metabolomic analyses revealed downregulation of coagulation–complement pathways and marked remodeling of lipid and lipoprotein networks. Importantly, these pathways are unlikely to operate independently. Chronic inflammation can directly perturb lipid metabolism through cytokine-mediated alterations in hepatic lipoprotein synthesis and clearance, while disrupted lipoprotein profiles may in turn modulate immune cell activation and inflammatory signaling. This bidirectional crosstalk may generate a self-reinforcing cycle of inflammatory and metabolic dysregulation^[10]^ and foster a pro-carcinogenic microenvironment. Mediation analyses further confirmed that inflammation and metabolic dysfunction played a moderate but biologically meaningful partial mediating role in this association. The remaining majority of the association may be attributable to other mechanisms, including additional inflammatory mediators, oxidative stress, immune dysregulation, and gut microbiota-related metabolites. Together, these findings support the view that the CKM–cancer relationship is more likely driven by acquired inflammatory–metabolic perturbations, highlighting potentially modifiable pathways for prevention. The particularly elevated risk observed in patients with concurrent cardiovascular and renal disease highlights a subgroup that may benefit from enhanced multidisciplinary surveillance. Future efforts integrating cardio-oncology principles may help refine risk stratification and guide preventive strategies targeting inflammatory and metabolic pathways.

## Supplementary Information

Supplementary materials are only available at the official site of the journal (www.intern-med.com).

## Supplementary Material

Supplementary Material Details
