# Supplementary material for: Cardiovascular-kidney-metabolic syndrome and cancer risk: Evidence from multi-omics analysis results
Source: J Transl Int Med. 2026 Aug 24;14(4):654–7. doi: 10.1515/jtim-2026-0072 (PMC13508829; doi:10.1515/jtim-2026-0072)
Supplement: Supplementary file 1 [file jtim-2026-0072_sm.pdf]

Supplementary materials

Supplementary Table S1: Participant descriptive characteristics at recruitment.

Characteristic	UK Biobank				US NHANES				CN CHARLS			
	**Total (*n* = 182,140)**	**Cancer-free participants (*n* = 163,750)**	**Participants with cancer (*n* = 18,390)[a]**	***P***	**Total (*n* = 18,097)**	**Cancer-free participants (*n* = 16,783)**	**Participants with cancer (*n* = 1314)**	***P***	**Total (*n* = 7944)**	**Cancer-free participants (*n* = 7811)**	**Participants with cancer (*n* = 133)[a]**	***P***
Age at recruitment, y												
30-44	17,608 (9.7)	16,939 (10.3)	669 (3.6)	< 0.001	5534 (30.6)	5412 (32.2)	122 (9.3)	< 0.001	161 (2.0)	159 (2.0)	2 (1.5)	0.889
45-49	23,207 (12.7)	22,001 (13.4)	1206 (6.6)		1852 (10.2)	1781 (10.6)	71 (5.4)		1481 (18.6)	1459 (18.7)	22 (16.5)	
50-54	27,411 (15.0)	25,373 (15.5)	2038 (11.1)		1816 (10.0)	1740 (10.4)	76 (5.8)		1188 (15.0)	1170 (15.0)	18 (13.5)	
55-59	33,037 (18.1)	29,796 (18.2)	3241 (17.6)		1544 (8.5)	1456 (8.7)	88 (6.7)		1721 (21.7)	1689 (21.6)	32 (24.1)	
≥ 60	80,877 (44.4)	69,641 (42.5)	11,236 (61.1)		7351 (40.6)	6394 (38.1)	957 (72.8)		3393 (42.7)	3334 (42.7)	59 (44.4)	
Sex												
Female	96,652 (53.1)	88,191 (53.9)	8461 (46.0)	< 0.001	9222 (51.0)	8491 (50.6)	731 (55.6)	< 0.001	4388 (55.2)	4318 (55.3)	70 (52.6)	0.602
Male	85,488 (46.9)	75,559 (46.1)	9929 (54.0)		8875 (49.0)	8292 (49.4)	583 (44.4)		3556 (44.8)	3493 (44.7)	63 (47.4)	
Ethnicity												
White	182,140 (100)	163,750 (100)	18,390 (100)	-	8431 (46.6)	7603 (45.3)	828 (63.0)	< 0.001	-	-	-	-
Not White	-	-	-		9666 (53.4)	9180 (54.7)	486 (37.0)		7944 (100)	7811 (100)	133 (100)	
Body mass index, kg/m^2												
< 18.5	867 (0.5)	793 (0.5)	74 (0.4)	< 0.001	221 (1.2)	198 (1.2)	23 (1.8)	0.184	503 (6.3)	496 (6.4)	7 (5.3)	0.617
18.5 ~ 24.9	58,520 (32.1)	53,366 (32.6)	5154 (28.0)		4724 (26.1)	4369 (26.0)	355 (27.0)		4908 (61.8)	4826 (61.8)	82 (61.7)	
25.0 ~ 29.9	78,347 (43.0)	70,190 (42.9)	8157 (44.4)		6431 (35.5)	5986 (35.7)	445 (33.9)		2124 (26.7)	2090 (26.8)	34 (25.6)	
≥ 30.0	44,406 (24.4)	39,401 (24.1)	5005 (27.2)		6721 (37.1)	6230 (37.1)	491 (37.4)		409 (5.1)	399 (5.1)	10 (7.5)	

Education level[b]												
Lower than high school	32,455 (17.8)	28,300 (17.3)	4155 (22.6)	< 0.001	5051 (27.9)	4713 (28.1)	338 (25.7)	0.257	7174 (90.3)	7058 (90.4)	116 (87.2)	0.010
High school or equivalent level	83,837 (46.0)	75,914 (46.4)	7923 (43.1)		4096 (22.6)	3781 (22.5)	315 (24.0)		544 (6.8)	537 (6.9)	7 (5.3)	
Higher than high school	64,350 (35.3)	58,230 (35.6)	6120 (33.3)		8931 (49.4)	8272 (49.3)	659 (50.2)		224 (2.8)	214 (2.7)	10 (7.5)	
Unknown	1498 (0.8)	1306 (0.8)	192 (1.0)		19 (0.1)	17 (0.1)	2 (0.1)					
Marital status												
Married	135,031 (74.1)	121,524 (74.2)	13,507 (73.4)	< 0.001	11,685 (64.6)	10,867 (64.8)	818 (62.3)	0.073	6758 (85.1)	6645 (85.1)	113 (85.0)	0.980
Not married	13,484 (7.4)	12,343 (7.5)	1141 (6.2)		6235 (34.5)	5748 (34.2)	487 (37.1)		1186 (14.9)	1166 (14.9)	20 (15.0)	
Unknown	33,625 (18.5)	29,883 (18.2)	3742 (20.3)		177 (1.0)	168 (1.0)	9 (0.7)		-	-	-	
Socioeconomic status[c]												
Low	38,271 (21.0)	34,343 (21.0)	3928 (21.4)	< 0.001	4690 (25.9)	4382 (26.1)	308 (23.4)	0.022	189 (2.4)	187 (2.4)	2 (1.5)	0.521
Medium	111,580 (61.3)	100,565 (61.4)	11,015 (59.9)		6270 (34.6)	5766 (34.4)	504 (38.4)		4134 (52.0)	4063 (52.0)	71 (53.4)	
High	32,068 (17.6)	28,642 (17.5)	3426 (18.6)		5550 (30.7)	5157 (30.7)	393 (29.9)		3518 (44.3)	3458 (44.3)	60 (45.1)	
Unknown	221 (0.1)	200 (0.1)	21 (0.1)		1587 (8.8)	1478 (8.8)	109 (8.3)		103 (1.3)	103 (1.3)	0 (0)	
Smoke												
Never	99,526 (54.6)	90,838 (55.5)	8688 (47.2)	< 0.001	9362 (51.7)	8796 (52.4)	566 (43.1)	< 0.001	4926 (62.0)	4847 (62.1)	79 (59.4)	0.549
Previous	63,943 (35.1)	56,608 (34.6)	7335 (39.9)		5096 (28.2)	4562 (27.2)	534 (40.6)		638 (8.0)	624 (8.0)	14 (10.5)	
Current	18,671 (10.3)	16,304 (10.0)	2367 (12.9)		3639 (20.1)	3425 (20.4)	214 (16.3)		2380 (30.0)	2340 (30.0)	40 (30.1)	
Alcohol												
Never	5553 (3.0)	4973 (3.0)	580 (3.2)	0.128	2320 (12.8)	2154 (12.8)	166 (12.6)	< 0.001	4738 (59.6)	4664 (59.7)	74 (55.6)	0.021
Current	17,0318 (93.5)	153,192 (93.6)	17,126 (93.1)		11,956 (66.1)	11,133 (66.3)	823 (62.6)		2581 (32.5)	2541 (32.5)	40 (30.1)	
Previous	6124 (3.4)	5455 (3.3)	669 (3.6)		2843 (15.7)	2570 (15.3)	273 (20.8)		625 (7.9)	606 (7.8)	19 (14.3)	
Unknown	145 (0.1)	130 (0.1)	15 (0.1)									
Diet point[d]												
Low	16,919 (9.3)	15,026 (9.2)	1893 (10.3)	< 0.001	4804 (26.5)	4489 (26.7)	315 (24.0)	0.054	-	-	-	-

Medium	163,379 (89.7)	147,045 (89.8)	16,334 (88.8)		4811 (26.6)	4469 (26.6)	342 (26.0)		-	-	-	
High	1842 (1.0)	1679 (1.0)	163 (0.9)		4805 (26.6)	4421 (26.3)	384 (29.2)		-	-	-	
Unknown	-	-	-		3677 (20.3)	3404 (20.3)	273 (20.8)		-	-	-	
Physical activity level[e]												
Low	25,684 (14.1)	22,969 (14.0)	2715 (14.8)	< 0.001	6306 (34.8)	5785 (34.5)	521 (39.6)	< 0.001	-	-	-	-
Moderate	71,084 (39.0)	64,103 (39.1)	6981 (38.0)		5227 (28.9)	4855 (28.9)	372 (28.3)		-	-	-	
High	44,932 (24.7)	40,500 (24.7)	4432 (24.1)		3668 (20.3)	3463 (20.6)	205 (15.6)		-	-	-	
Unknown	40,440 (22.2)	36,178 (22.1)	4262 (23.2)		2896 (16.0)	2680 (16.0)	216 (16.4)		-	-	-	
CKM stage												
0	13,422 (7.4)	12,547 (7.7)	875 (4.8)	< 0.001	1371 (7.6)	1300 (8.0)	71 (3.9)	< 0.001	823 (10.4)	811 (10.4)	12 (9.0)	0.155
1	10,481 (5.8)	9704 (5.9)	777 (4.2)		2641 (14.6)	2523 (15.5)	118 (6.5)		901 (11.3)	891 (11.4)	10 (7.5)	
2	103,740 (57.0)	95,225 (58.2)	8515 (46.3)		7168 (39.6)	6790 (41.7)	378 (20.7)		2974 (37.4)	2929 (37.5)	45 (33.8)	
3	38,744 (21.3)	32,681 (20.0)	6063 (33.0)		4604 (25.4)	3788 (23.3)	816 (44.8)		2232 (28.1)	2191 (28.1)	41 (30.8)	
4	15,753 (8.6)	13,593 (8.3)	2160 (11.7)		2313 (12.8)	1874 (11.5)	439 (24.1)		1014 (12.8)	989 (12.7)	25 (18.8)	
Leukocyte, mean (SD), 10^9 cells/L	6.89 (1.93)	6.86 (1.85)	7.14 (2.50)	< 0.001	6.80 (2.44)	6.78 (2.17)	7.10 (4.64)	< 0.001	-	-	-	-
Lymphocyte, mean (SD), 10^9 cells/L	1.95 (0.94)	1.94 (0.82)	2.02 (1.68)	< 0.001	2.01 (1.34)	2.00 (0.77)	2.14 (4.13)	< 0.001	-	-	-	-
Monocyte, mean (SD), 10^9 cells/L	0.48 (0.21)	0.48 (0.20)	0.50 (0.24)	< 0.001	0.54 (0.20)	0.53 (0.20)	0.57 (0.26)	< 0.001	-	-	-	-
Neutrophil, mean (SD), 10^9 cells/L	4.25 (1.40)	4.23 (1.39)	4.39 (1.51)	< 0.001	4.01 (1.72)	4.00 (1.73)	4.13 (1.61)	0.009	-	-	-	-
CRP, mean (SD), mg/L	2.56 (4.23)	2.52 (4.19)	2.91 (4.52)	< 0.001	1.43 (4.65)	1.39 (4.53)	1.91 (5.94)	< 0.001	-	-	-	-
NLR, mean (SD)	2.37 (1.24)	2.37 (1.24)	2.43 (1.24)	< 0.001	2.21 (1.21)	2.19 (1.17)	2.56 (1.65)	< 0.001	-	-	-	-
SII, mean (SD)	604.28 (365.81)	603.02 (364.65)	615.56 (375.76)	< 0.001	550.07 (409.99)	545.18 (408.59)	612.84 (422.75)	< 0.001	-	-	-	-

[a] except for non-melanoma skin cancer. [b] The education level in UK Biobank is classified as follows: college or university degree and other professional qualifications are classified as Higher than high school, A levels/AS levels or equivalent, O levels/GCSEs or equivalent, CSEs or equivalent, NVQ or HND or HNC or equivalent are classified as High school or equivalent level, none of the above is classified as Lower than high school. [c] Socioeconomic status in the UK Biobank was measured by Townsend deprivation index categorized into 5 quintiles: low (Q1), medium (Q2–Q4), high (Q5); Socioeconomic status in the NHANES was measured by family income to poverty ratios and is divided into three categories: high ($\geqslant$ 3.5), medium (> 1.3 to < 3.5), and low ($\leqslant$ 1.3); Socioeconomic status in the CHARLS was measured by self-reported standard of living. [d] Diet points in the UK Biobank cohort was measured by the methods developed by Lourida et al.and was categorized into 3 levels: low (0-1 points), medium (2-5 points), high (6-7 points); Diet points in the NHANES cohort was measured by HEI 2020 and was divided into three categories: low (Q1), medium (Q2), and high (Q3). [e] Physical activity level was classified as low (< 600 metabolic equivalent (MET) minutes/week), moderate (600 to 3000 MET-minutes/week), and high ($\geqslant$ 3000 MET-min/week). NHANES: National Health and Nutrition Examination Surveys; CHARLS: China Health and Retirement Longitudinal Study; GCSEs: General Certificate of Secondary Education; CSEs: Certificate of Secondary Education; HEI: Healthy Eating Index; CRP: C-reactive protein; NLR: Neutro-lymphocyte ratio; SII: Systemic Immune Inflammatory index; Q: quintile; SD: standard deviation.
